# THE CARE NAVIGATOR ROLE IN RURAL PRIMARY CARE MEMORY CLINICS

**DOI:** 10.1093/geroni/igae098.2525

**Published:** 2024-12-31

**Authors:** Debra Morgan, Melanie Bayly, Julie Kosteniuk

**Affiliations:** University of Saskatchewan, Saskatoon, Saskatchewan, Canada; University of Saskatchewan, Saskatoon, Saskatchewan, Canada; University of Saskatchewan, Saskatoon, Saskatchewan, Canada

## Abstract

Collaborative dementia care models that include a navigator role achieve better outcomes but little is known about impacts in rural settings. Rural primary healthcare memory clinic teams in Saskatchewan include Coordinators from the Alzheimer Society of Saskatchewan (ASOS) First Link Program, which aims to connect persons living with dementia (PLWD) and families with supports at diagnosis. We examined perceptions of First Link Coordinators (FLC) and memory clinic team members about the FLC role, and evaluated impact of the role within the memory clinic (compared to self- and direct-referrals from other healthcare providers) on timeliness, number of completed FLC-client contacts, topics discussed, duration, and contact method. The QUAL+QUAN design included: (1) semi-structured interviews with three FLC and six team members in four rural memory clinics, (2) routinely collected ASOS client data. Separate inductive thematic analyses with FLC and team interviews (Jan 2021-Apr 2023) identified two common themes: benefits for PLWD and families (eg, early and ongoing connection to emotional support) and benefits for team members (eg, can focus on their unique role). Face-to-face contact established a FLC-family relationship and facilitated follow-up contact. Longitudinal retrospective analysis of ASOS data found that between Dec 2017-Dec 2021, 139 clients (predominantly spouses, children) were referred. Compared to other referrals, statistically significant differences (p<.05) included: memory clinic clients contacted sooner after referral, longer duration of first contact, more completed contacts, more topics discussed. Findings demonstrate the added value of FLC in rural memory clinics. Future research should explore long-term outcomes for PLWD and families.

